# Rad9 modulates the *P21*^*WAF1 *^pathway by direct association with p53

**DOI:** 10.1186/1471-2199-8-37

**Published:** 2007-05-21

**Authors:** Kazuhiro Ishikawa, Hideshi Ishii, Yoshiki Murakumo, Koshi Mimori, Masahiko Kobayashi, Ken-ichi Yamamoto, Masaki Mori, Hiroshi Nishino, Yusuke Furukawa, Keiichi Ichimura

**Affiliations:** 1 Department of Otolaryngology-Head and Neck Surgery, Jichi Medical University School of Medicine, Tochigi, Japan; 2 Center for Molecular Medicine, Jichi Medical University School of Medicine, Tochigi, Japan; 3 Department of Pathology, Nagoya University Graduate School of Medicine, Aichi, Japan; 4 Institute of Bioregulation, Kyushu University, Ohita, Japan; 5 Kanazawa University Cancer Research Institute, Ishikawa, Japan

## Abstract

**Background:**

Previous studies suggest that human *RAD9 *(hRad9), encoding a DNA damage checkpoint molecule, which is frequently amplified in epithelial tumor cells of breast, lung, head and neck cancer, participates in regulation of the tumor suppressor p53-dependent transactivation of pro-survival *P21*^*WAF1*^. This study examined the exact mechanism of the hRad9 function, especially through the phosphorylation of the C-terminus, in the transcription regulation of *P21*^*WAF1*^.

**Results:**

The transfection of phosphorylation-defective *hRAD9 *mutants of C-terminus resulted in reduction of the p53-dependent *P21*^*WAF1 *^transactivation; the knockdown of total hRad9 elicited an increased *P21*^*WAF1 *^mRNA expression. Immunoprecipitation and a ChIP assay showed that hRad9 and p53 formed a complex and both were associated with two p53-consensus DNA-binding sequences in the 5' region of *P21*^*WAF1 *^gene. The association was reduced in the experiment of phosphorylation-defective *hRAD9 *mutants.

**Conclusion:**

The present study indicates the direct involvement of hRad9 in the p53-dependent *P21*^*WAF1 *^transcriptional mechanism, presumably via the phosphorylation sites, and alterations of the hRad9 pathway might therefore contribute to the perturbation of checkpoint activation in cancer cells.

## Background

DNA damage checkpoints are signal transduction pathways that maintain the proper order of cell cycle events. When DNA is damaged or perturbed during replication, the cells respond by the activation of evolutionarily conserved signal transduction pathways that delay the progression of the cell cycle and induce repair of the damaged DNA. These signal transduction pathways include protein sensors that recognize aberrant DNA structures and activate kinases, thereby inducing phosphorylation cascades that ultimately lead to cell cycle arrest and DNA repair [1,2]. Failure of this cell cycle surveillance mechanism can cause genomic instability which eventually leads to the formation of cancer in mammals [3].

hRad9 protein is the human homologue of *Schizosaccharomyces pombe *Rad9, a member of the checkpoint *rad *genes (*rad1+*, *rad3+*, *rad9+*, *rad17+*, *rad26+*, and *hus1+*) which are required for the S phase (DNA replication) and G2 (DNA mitosis) check points [4,5]. Like its yeast counterpart, hRad9 forms a ring-shaped, heterotrimeric complex with the hRad1 and hHus1 proteins [6,7]. Each member of hRad9-hRad1-hHus1 complex (also known as the 9-1-1 complex), shares sequence homology with proliferating cell nuclear antigen (PCNA), a homotrimer that encircles the DNA and tethers DNA polymerase δ during DNA synthesis [7-10]. PCNA is loaded onto DNA by the pentameric protein complex replication factor C (RFC) [11], which is composed of one large subunit and four smaller subunits. In a manner analogous to PCNA and RFC, 9-1-1 complex is loaded onto DNA by a complex between hRad17 and the four small subunits of RFC [12]. Since DNA damage induces hRad17-dependent association of 9-1-1 complex with chromatin, the 9-1-1 complex is believed to be involved in the direct recognition of DNA lesions during the initial stages of the checkpoint response; the 9-1-1 complex may thus be associated with chromatin following DNA damage to transduce signals for DNA damage-activated checkpoint signaling pathways [13]. In mammalian cells, the signal initiated by the sensors, two phosphatidylinositol 3-kinase-related kinases (PIKK), ataxia-telangiectasia mutated (ATM) and ataxia-telangiectasia mutated and Rad3-related (ATR), plays a central role in the checkpoint signaling pathways [14]. ATM and ATR are activated by downstream signaling proteins, genotoxins and phosphorylation including Chk1 and Chk2, two protein kinases that regulate checkpoint responses [15-17]. hRad9 is highly modified by phosphorylation in at specific points of the cell cycle after DNA damage, and it also plays a critical role in checkpoint signaling [18]. ATM-mediated phosphorylation at Ser-272 of hRad9 is required for IR-induced G1/S checkpoint activation [19,20]; Other phosphorylation sites in the C-terminal region are also essential for Chk1 activation following hydroxyurea (HU), IR, and UV treatment [20], although the exact function of hRad9 in cell cycle control has not yet been completely characterized.

The tumor suppressor gene *TP53 *controls cell cycle checkpoints, apoptosis, and genomic stability [21]. A defect in the pathway, involving p53, is essential for the malignant progression of cancer [22]. When cells with wild-type *TP53 *are exposed to DNA-damaging agents, p53 is functionally activated, p53 protein level rises, and p53 binds to and transcriptionally activates the promoters of target genes [21,23,24]. These target genes include; *P21*, *MDM2*, *GADD45*, *BAX*, *IGF-BP3 *and *CYCLIN G*. The *P21 *gene product was originally identified, as a potential target for the p53 tumor suppressive activity (WAF1) [25]. It is either an inhibitor of the G1 cyclin-dependent kinases (Cip1) [26], or an inhibitor of DNA synthesis that is expressed during cellular senescence (SDI1) [27]. It is known that p21 is a major effector of the G1 cell cycle checkpoint. Therefore, p53 is a negative regulator of the cell cycle progression and it controls the transition from G1 to S phase of the cell cycle [28]. This report demonstrates that hRad9 plays a role in the modulation of *P21 *transcription by direct interaction with p53. Furthermore, the substitution of the phosphorylation sites on hRad9 to Ala resulted in an alteration of the regulation of *P21*. The present study supports the hypothesis that hRad9 plays a role in the modulation of *P21 *transcription, presumably via competition with p53, that involves its C-terminus, which would be essential for the cellular response to DNA damage.

## Results and discussion

### P21 is activated immediately after UV treatment

**Figure 1:**
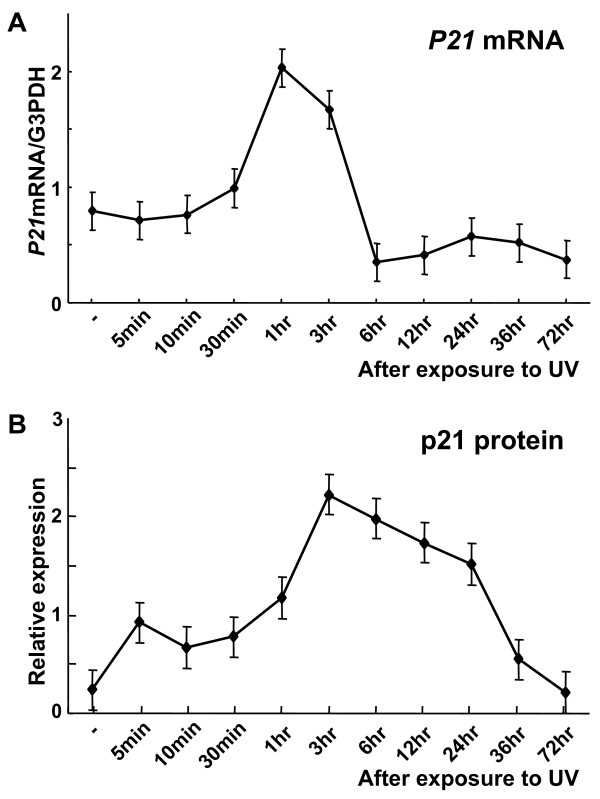
**UV-induced effect on the expression of *P21 *mRNA and p21 protein**. 293 cells were exposed to UV at 20 J/m^2 ^and harvested 0 to 72 hr after treatment as indicated. Data represent the means ± SD of three independent experiments. (A) The time course of *P21 *mRNA expression. RNA was extracted for RT-PCR. The ratios of *P21 *mRNA to *G3PDH *mRNA were measured with densitometry. (B) The time course of p21 protein expression. Cell lysates were extracted and separated by SDS-PAGE, transferred to a nitrocellulose membrane, and probed with anti-p21 antibody. The signal intensity of the Western blotting is shown.

Several previous studies have shown that genotoxic stress induces the stabilization and transient accumulation of wild-type p53 protein in mammalian cells, leading to an increase of expression of p53 down-stream genes, including *P21 *[21,25]. Employing RT-PCR, the level of *P21 *mRNA expression of human embryonic kidney 293 cells was examined in these experiments. The level of *P21 *mRNA expression substantially increased at 30 min after UV irradiation and reached maximal levels at 1 hr thereafter. At that time, the mRNA levels were reduced but still detectable until 72 hr after UV exposure (Fig. 1A). Next, using the protein equivalent to each time points, a Western blotting analysis was performed to examine the time course of p21 protein expression. As shown in Figure 1B, a substantial increase of p21 was seen at 3, 12, and 24 hr following exposure to UV. These results indicate that the accumulation of both *P21 *transcript and p21 protein occurred, and G1/S checkpoint was activated after UV irradiation. Since 293 cells are immortalized by E1B, derived from human adenoviral proteins, and might be defective in p53 signaling, another MRC5 fibroblast cell line with wild-type p53 was examined, and observed that *P21 *mRNA increased about 3.3 fold after UV irradiation (Additional file 1), thus suggesting that exposure to UV leads an increase of *P21 *expression of both cell lines similarly under these conditions. This expression pattern of *P21 *mRNA and p21 protein suggests that the G1/S checkpoint is activated immediately after DNA damage, and inhibits damaged cells from progressing through the cell cycle and entering the S phase [29]. After the peak of activation, *P21 *transcription declined as observed in a recent report that indicated that p21 can be degraded during excision repair [30]. This suggests that more than the arrest of the cell cycle at G1 or a broad time period could thus be involved in the decrease of *P21 *mRNA and protein following UV exposure.

### The role of hRad9 in p53-dependent P21 activation through its C-terminus

**Figure 2:**
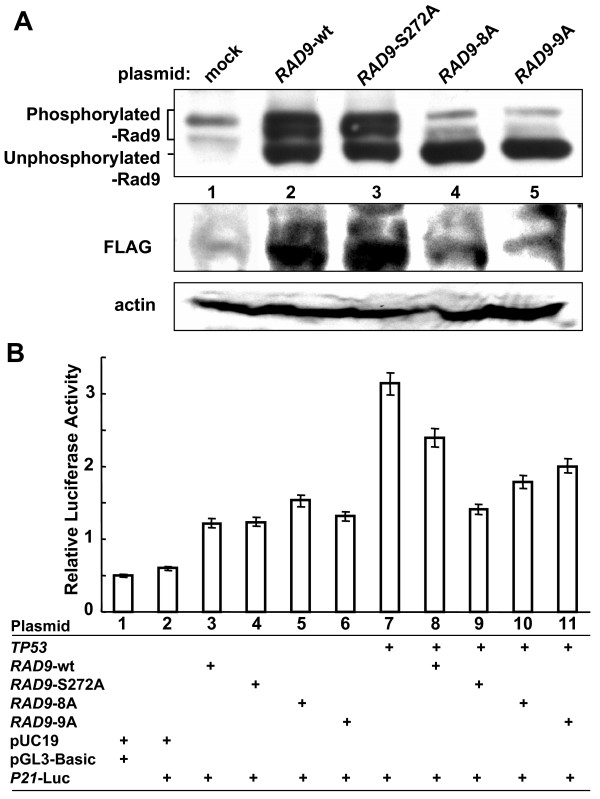
**Effect of wild-type and phosphorylation-defective hRad9 mutants expression on *P21 *transactivation**. (A) Western blots of lysates from 293 transfectants of wild-type or phosphorylation-defective *RAD9 *mutants. Lysates were analyzed by Western blotting, probed with anti-Rad9 (*top*), anti-FLAG (*middle*), or anti-actin (*bottom*) antibody. (B) *P21 *transcription assay with wild-type and phosphorylation-defective *RAD9*. mutants using *P21 *promoter-luciferase reporter system. After transfection into 293 cells, the relative luciferase activities are shown as the ratios of *Firefly *to *Renille*. Data represent the means ± SD of four independent experiments.

Utilizing the *P21 *promoter-luciferase reporter system, the functional aspect of regulation of *P21 *by hRad9 was assessed. Since phosphorylation of hRad9 is required for DNA damage checkpoint activation [20], the requirement of phosphorylation of hRad9 for *P21 *transcription was investigated using wild-type and the following phosphorylation-defective *RAD9 *mutants: 1) *RAD9*-S272A, with a substitution of Ser-272 with an Ala residue, 2) *RAD9*-9A, with substitutions of C-terminus phosphorylation sites including Ser-272, Ser-277, Ser-328, Ser-336, Ser-341, Ser-355, Ser-375, Ser-380, and Ser-387, with Ala, and 3) *RAD9*-8A, with substitutions of all C-terminus phosphorylation sites except for Ser-272, with Ala. Western blotting was performed to confirm the expression of the protein in wild-type and mutants,. Wild-type Rad9 was detectably expressed as a series of large proteins, suggesting the phosphorylated form(s) of hRad9 (Fig. 2A, lane 2) [18]. Rad9-S272A also expressed with a similar series of large proteins, presumably corresponding to different phosphorylated species of hRad9, with a gradient of phosphorylation excluding the Ser272 residue. (Fig. 2A, lane 3) [18]. The transfection of *RAD9*-8A or *RAD9*-9A resulted in a reduced phosphorylation compared to wild-type Rad9 (Fig. 2A, lanes 4 and 5, compared with lane 2). The results indicate that phosphorylation-defective mutants are substantially expressed and retain the capacity for reduced phosphorylation [18]. Afterward, the promoter region of *P21 *was fused to a luciferase reporter (WWP-Luc-*P21 *promoter) and it was cotransfected along with plasmids into cells as indicated. Background levels of expression were low, as demonstrated by a control transfection of empty vector and promoter-less pGL3-basic (Fig. 2B, column 1). By using the reporter vector, containing the p53-binding consensus sequence [31], the introduction of p53 expectedly induced luciferase activity (Fig. 2B, column 7). The introduction of the wild-type Rad9 also induced the positive luciferase activity (Fig. 2B, column 3), although not as intensively as p53. The co-expression of p53 and wild-type Rad9 induced luciferase activity (Fig. 2B, column 8) at an intermediate level in comparison to that seen when each protein is transfected independently. Considering these data, in association with the previous report of the potential transactivating property of hRad9 for the *P21 *promoter [32], these results support the concept that hRad9 can stimulate the transcription of *P21 *and it may modulate the p53 function. The luciferase activity was comparable among wild-type and phosphorylation-defective mutants following the plasmid introduction (Fig. 2B, columns 4 to 6, compared with 3), and was lower in the co-expression of mutants (Fig. 2B, columns 9 to 11, compared with 8), suggesting that the phosphorylation of hRad9 is involved in regulation of p53-dependent *P21 *transcription. Yin *et al*. [32] reported that wild-type hRad9 activates *P21 *transcription and that the co-expression of p53 and wild-type hRad9 results in the intermediate transcriptional activity by p53 or hRad9 alone, consistent with the present results. This suggests that phosphorylation may thus play a possible role in p53 dependent *P21 *transcription.

### Knock down of endogenous hRad9 increases the transcription of P21

**Figure 3:**
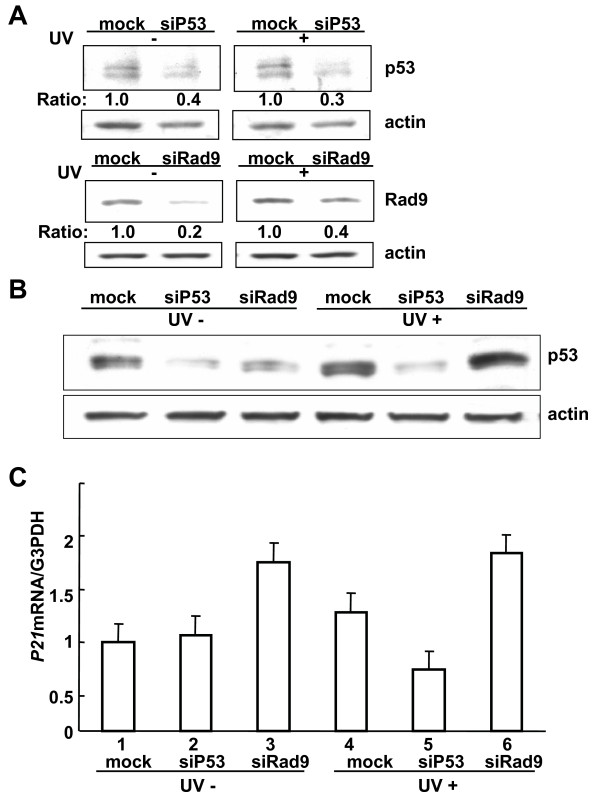
**Knockdown experiment of hRad9 and p53 using siRNAs**. (A) hRad9 and p53 expression after transfection with *hRAD9 *(siRad9) or *TP53 *siRNA (siP53). The signals of immunoblots were measured with a densitometer, and the ratios of hRad9 or p53 to actin control were depicted in the figure. (B) p53 expression after transfection with *hRAD9 *(siRad9) or *TP53 *siRNA (siP53). (C) Alteration of *P21 *mRNA expression in the knockdown experiment by *hRAD9 *or *TP53 *siRNA. Cells were treated with *hRAD9 *(siRad9) or *TP53 *siRNA (siP53) and subjected to UV exposure or mock treatment. The cells were harvested and RNAs were extracted for RT-PCR with *P21 *primers. The relative amount of *P21 *mRNA to *G3PDH *mRNA is shown. Data represent the means ± SD of three independent experiments.

Endogenous hRad9 was knocked down using siRNA to address the role of hRad9 for the transcription of *P21 *in response to UV exposure. A Western blot analysis showed that siRNA treatment resulted in the reduction of endogenous p53 or hRad9 (Fig. 3A). In addition, hRad9 siRNA did not increase p53 protein compared to mock treatment (Fig. 3B). Using RT-PCR, the *P21 *mRNA level was determined. The knockdown of *hRAD9 *resulted in an increase in the level of *P21 *mRNA (Fig. 3C, column 3), whereas the knockdown of *TP53 *showed levels of *P21 *mRNA comparable to mock-treatment (Fig. 3C, column 2, compared with 1). This suggests that hRad9 plays a role in modulating *P21 *transactivation. After UV treatment, the mock-treated cells showed an increase of *P21 *mRNA (Fig. 3C, column 4), compared with UV (-) control, under these conditions. *TP53 *siRNA transfection resulted in an obvious reduction of *P21 *mRNA (Fig. 3C, column 5), thus suggesting that the effect of p53 reduction was appreciable after UV-induced damage under those conditions. In contrast, *hRAD9 *knockdown resulted in an apparent increase of *P21 *mRNA after UV exposure (Fig. 3C, column 6). The UV treatment was not simply additive to the *hRAD9 *knockdown in *P21 *transactivation, thus suggesting that the reduction of endogenous hRad9 resulted in a profound effect in UV damage-dependent and -independent *P21 *pathway. Furthermore, semi-quantitative RT-PCR and PCR-Southern blotting was performed to confirm the effect of Rad9 siRNA, and similar results were obtained (Additional files 2A and 2B). Using *TP53*-deficient MEFs, real time RT-PCR was used to confirm the effect of knockdown of Rad9 in *P21 *transcription, and this demonstrated an increase in *P21 *mRNA after knockdown of Rad9 (Additional file 2C), thus supporting the concept that Rad9 is not only an activator, but also a modulator in this pathway. The transactivation of *P21 *in the absence and reduction of the p53 and Rad9 may be due to transcription factors, such as sp1 [33]. In addition, a p53-deficient cell line TE-7, with transcriptionally inactive *TP53 *[34], was used to study the effect of the introduced hRad9 and p53 proteins under p53 negative background. TE-7 cells were transfected with wild-type or phosphorylation-defective mutant *RAD9*, and *TP53 *plasmids, and the expression of each protein was confirmed by Western blotting; the exogenous expression of wild-type and mutant *RAD9 *elicited the induction of *P21 *mRNA and its product, associated with Ser15 phosphorylation of transfected p53, whereas endogenous p53 and its phosphorylated form were undetectable without transgene introduction (Additional file 3A, 3B, and data not shown), thus suggesting that Rad9 may play a role in the p53-dependent p21 transactivation and Rad9-p53 might express a certain active function in p53 negative cancer cells, compatible with the previous report [32]. One might speculate that Rad9-p53 has a high affinity for the p21 promoter; rather than the induction of p21, the transfection could result in the reduction of p21 in endogenous p53-positive cells, and might stimulate p21 in p53-negative background. It also should be noted that numerous or uncharacterized, additional alterations might accumulate in cancer-derived cell lines. Taken together, the present data indicate that the transfection of hRad9 plays a role in *P21 *transcription, depending on the co-expressed p53 (Fig. 2), and the knockdown of hRad9 can stimulate *P21 *transactivation, thus suggesting that the protein level of hRad9 may be involved in modulation and regulation of *P21 *transactivation. Considering that other studies show that hRad9 accumulation after DNA damage [12,13] may enhance the modulation of *P21 *promoter activation, this supports the hypothesis that hRad9 may regulate *P21 *transcription in concert with p53, and that the reduction of hRad9 might elicit the checkpoint response. Recent studies with tumors indicate that the *hRAD9 *gene is located in a chromosomal region at 11q13. This genomic region is amplified and both mRNA and protein are frequently overexpressed in breast, lung, head and neck cancer [35,36]. This up-regulation is correlated with tumor size and local recurrence [35]. Furthermore, silencing *hRAD9 *by RNA interference inhibits cell proliferation *in vitro *[35]. These observations are consistent with the findings of the present study. Nevertheless, there is still insufficient data to determine whether hRad9 might be involved directly or indirectly in p53-dependent *P21 *activation. Therefore, the assessment of the direct association of hRad9-p53 is the next step.

### hRad9 associates with p53

**Figure 4:**
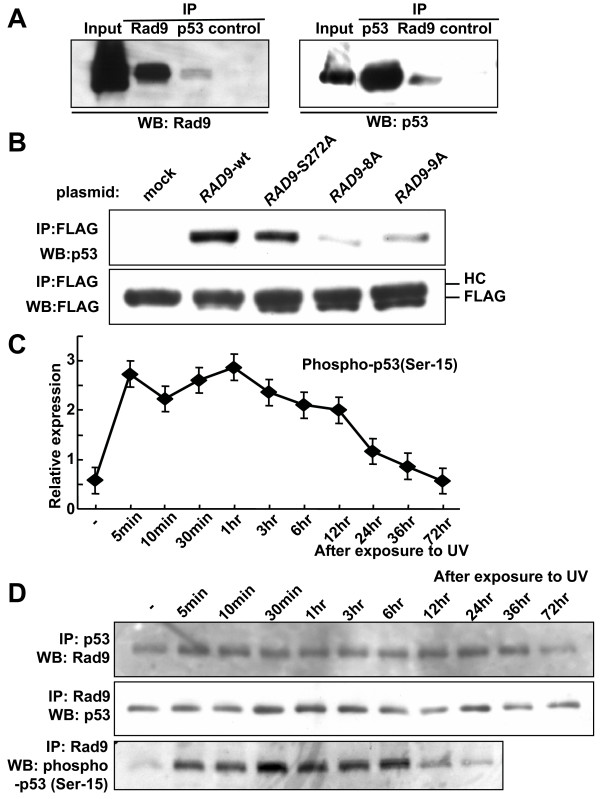
**Interaction between hRad9 and p53**. (A) Endogenous association of hRad9 and p53. Immunoprecipitation and Western blot analysis were performed using lysates of 293 cells. Anti-c-kit antibody was used as a negative control. (B) Immunoprecipitation using cell lysates of 293 transfectants with FLAG-tagged wild-type or phosphorylation-defective *RAD9 *plasmids. Each cell lysate was immunoprecipitated with anti-FLAG antibody and immunoblotted with anti-p53 (*top*) or anti-FLAG (*bottom*) antibody. HC, IgG heavy chain of the anti-FLAG antibody. (C) The time course experiment for the accumulation of phosphorylated p53 at Ser-15 after UV. 293 cells were exposed to UV at 20 J/m^2 ^and harvested 0 to 72 hr after treatment as indicated. A Western blot analysis was performed using anti-phosphorylated p53 (Ser-15) antibody. Relative expression of phosphorylated p53 to whole p53 is shown. Data represent the means ± SD of three independent experiments. (D) The time course experiment of hRad9/p53 complex formation after exposure to UV. 293 cells were exposed to UV at 20 J/m^2 ^and harvested 0 to 72 hr after treatment. Top, immunoprecipitation with anti-p53 antibody and Western blot analysis using anti-Rad9 antibody; middle, immunoprecipitation with anti-Rad9 antibody and Western blot with anti-p53 antibody; bottom, immunoprecipitation with anti-Rad9 antibody and Western blot with anti-phosphorylated p53 (Ser-15) antibody.

The data reported above indicate that transfection of hRad9 modulates p53-dependent *P21 *promoter activation, and that knockdown of hRad9 stimulates p53-dependent *P21 *promoter activation. Therefore, the association of endogenous hRad9 with endogenous p53 was examined using immunoprecipitation. Endogenous hRad9 was co-immunoprecipitated with p53 from 293 cells (Fig. 4A). The hRad9-p53 association was then assessed in another MRC5 cells using immunoprecipitation and similar results were obtained (Additional file 4), thus suggesting that the association is not specific to the cell type. Considering these results, along with those of the pull-down assay of GST-fusion p53 with [^35^S] methionine-labeled *in vitro*-translated hRad9 protein (data not shown), the findings of the present study strongly suggests that hRad9 acts as a modulator of *P21 *transcription through a direct association with p53. To investigate whether phosphorylation of hRad9 affects the binding to p53, wild-type or phosphorylation-defective *RAD9 *mutants were transfected, and immunoprecipitation was performed. The results showed p53 to be associated with hRad9, in both the wild-type and phosphorylation-defective mutants (*RAD9*-S272A, *RAD9*-8A, and *RAD9*-9A). However the association with *RAD9*-8A or *RAD9*-9A was somewhat reduced (Fig. 4B). The data implicate the substantial association of p53 with intact hRad9, and that the hRad9 mutants might be altered in capacity for p53 binding, presumably due to conformational changes and affinity of complex, which would result the release of immunoprecipitated components after extensive washes.

Previous studies have showed that p53 protein can be phosphorylated at Ser-15 within 1 hr after DNA damage [37]. A Western blot analysis indicated that Ser-15 of p53 was phosphorylated 5 min after UV treatment in the present experiments (Fig. 4C). The time course of this reaction was examined to determine whether the association of hRad9 and p53 might be altered after UV irradiation. Figure 4D demonstrates that the phosphorylation of p53 at Ser-15 in the immunoprecipitated components increased at 5 min and reached a maximal level at 6 hr after UV treatment, and declined, consistent with the Western blot findings in Figure 4C, whereas the total amount of Rad9-p53 interaction did not increase. In addition, other phosphorylation sites of p53, including Ser-6, Ser-9, Ser-20, Ser-37, Ser-46, and Ser-392 were also examined and they were phosphorylated temporally, regardless of the positive association of hRad9 with p53 over the time course, as observed Ser-15 (Fig. 4D, and data not shown). These results indicate that the binding of hRad9 and p53 is not significantly affected by phosphorylation, though it is possible that the modifications of the phosphorylation in these amino acid residues are not involved in the binding of the components.

The present immunoprecipitation study revealed hRad9 to be associated with p53, and the association was detected regardless of degree of phosphorylation of p53 and presumably of hRad9. A previous report also demonstrated that constitutive phosphorylation of hRad9 does not influence the stability of the 9-1-1 complex [20]. It is suggested that hRad9, as a complex with p53, may be involved in the transactivation of *P21 *and that the phosphorylations of hRad9 and p53 might modulate the transactivation activity of the complex. Whereas none of the phosphorylation sites of hRad9 targeted in the present study have been previously reported to be required for genotoxin-induced chromatin binding [20], the present data suggest that hRad9 phosphorylation might be involved in binding affinity for p53-consensus binding sites.

### Phosphorylation of hRad9 affects the preference of p53 for binding sites

**Figure 5:**
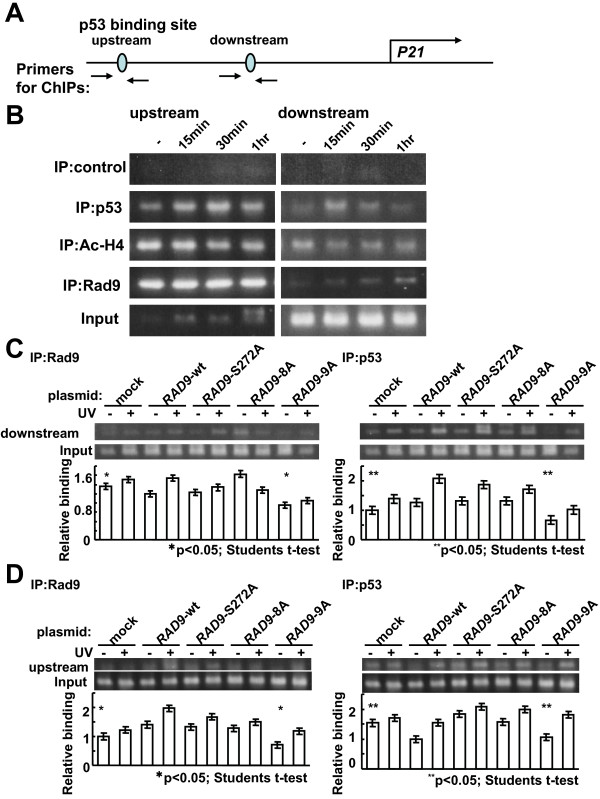
**UV exposure-dependent increase of the hRad9 association to the p53-binding site in the *P21 *promoter**. (A) Schematic representation of the *P21 *promoter showing the location of the oligonucleotide primer sets, utilized in the ChIP assays. (B) ChIP assay with anti-p53 antibody, anti-acetylated histone H4 antibody or anti-Rad9 antibody for upstream (*left*) and downstream (*right*) *P21 *promoter sites. 293 cells were harvested with or without exposure to UV. ChIP assays were performed using anti-p53 (p53) or anti-acetylated histone H4 antibody (Ac-H4). Anti-c-kit antibody was used as a negative control. (C) ChIP assay for the downstream *P21 *promoter site. 293 cells were transfected with wild-type or phosphorylation-defective *RAD9 *plasmids, and harvested before and after exposure to UV. ChIP assays were performed with anti-Rad9 (*left*) or anti-p53 antibody (*right*). The relative intensity of signals of immunoprecipitated DNA to input DNA was measured with densitometry. Data represent the means ± SD of three independent experiments. (D) ChIP assay for the upstream *P21 *promoter site. ChIP assays were performed with anti-Rad9 (*left*) or anti-p53 antibody (*right*) for *P21 *promoter upstream-binding site. Data represent the means ± SD of three independent experiments.

The effects of alterations of the affinity of hRad9/p53 for p53-consensus binding sites of *P21 *promoter for chromatin remodeling after UV treatment were investigated. Previous studies show that hRad9 specifically binds to a p53-consensus DNA-binding sequence in the *P21 *promoter and regulates *P21 *at the transcriptional level [32]. A ChIP assay was used to evaluate whether the affinity of hRad9/p53 complex for p53 binding sites may increase after UV treatment, and whether the phosphorylation of hRad9 affects the affinity for binding sites. The human *P21 *promoter contains two p53-binding sites (Fig. 5A), and the treatment with 5-fluorouracil significantly enhances the recruitment of p53 protein to both upstream and downstream *P21 *promoter regions [33]. Therefore, each of the two p53-binding sites was observed. A ChIP assay with anti-p53 and anti-Rad9 antibodies showed that their binding to each of upstream and downstream *P21 *promoters was increased 15 to 30 min after UV treatment, whereas ChIP with anti-acethylated histone H4 antibody indicated that the acetylation around the *P21 *promoter was not altered significantly in the those conditions (Fig. 5B), thus suggesting that the association of p53 and hRad9, and its subsequent complex with *P21 *promoters is correlated with the regulation of transcription after UV exposure. Next, ChIP assays were performed with transfectants of the wild-type or phosphorylation-defective *RAD9 *plasmids. As shown in Figure 5C, hRad9 and p53 binding to the downstream site was inhibited by introduction of phosphorylation-defective mutants (as shown as asterisks). The association was increased after UV treatment, in all cases except for cells transfected with the *RAD9*-8A plasmid which showed a low affinity to the binding site. This suggests the possibility that systematic, scheduled or sequential phosphorylation of Ser residues in hRad9 might be necessary for efficient binding to p53-consensus DNA sequences. ChIP binding of hRad9 seems to be reduced slightly, compared to that of p53, which might be due to the specificity of the antibodies. Similar results with hRad9 and p53 were obtained at the upstream binding site (Fig. 5D). The present results suggest that the phosphorylation of the C-terminal region of hRad9 may play a role in modulation of the affinity for binding its consensus sites.

Recent studies have demonstrated the multifunctional roles of hRad9 a DNA damage sensor in the 9-1-1 complex, a G2/M checkpoint via the phosphorylation of Chk1, in DNA repair via DNA polymerase β [38] or flap endonuclease 1 [39], and in apoptosis via potential binding to Bcl-2 or Bcl-xL [40]. The present study, demonstrated the direct association of Rad9-p53 and the regulatory role of phosphorylation in the activation of *P21 *transcription, thus indicating that hRad9 is an important modulator, but not a unique player with a single function.

## Conclusion

hRad9 has a complex role in response to DNA damage, acting not only as an activator but also as a modulator in *P21 *transcription and contributing to the regulation of genomic integrity. If hRad9 was regulated inaccurately, the *P21 *could not regulate the appropriate G1/S transition and replication, thus resulting in the occurrence of unscheduled replications after DNA damage. Such events can contribute to the accumulation of pathological conditions and genomic instability in carcinogenesis and tumor progression.

## Methods

### Cell culture

Human embryonic kidney 293 cells, immortalized by E1B protein, derived from a part of the human adenovirus, and MRC5 fibroblasts with wild-type p53 status were cultured in DMEM with 10% fetal cow serum (FCS). TE-7 cell line derived from human esophageal cancers, which p53 is inactivated by transcriptional repression [34], was used. Murine embryonic fibroblast (MEF) cell lines were obtained from *TP53*-deficient and control wild-type mice (Jackson Laboratory). After pairs of mice were mated, MEFs obtained from sub-cutaneous tissue of embryos at 13.5 post-coital days were grown in DMEM medium with 20% FBS. Cells were transfected with 3 ug of expression plasmid of siRNA to inhibit Rad9 (pBAsi-mU6 plasmid, TAKARA, sequence in Table 1), selected in medium with 1 ug/ml puromycin. For exposure to UV, 60% to 70% confluent monolayer cells were washed and irradiated with UVC emitted by germicidal lamps (GL-15; NIPPO Electronic). Irradiation dose was measured with a digital UV-C densitometer (UCV-254; Custom). Control cells were taken into the UV exposure source but were not irradiated.

### Antibodies and plasmids

The following antibodies were used: anti-Rad9 (Alexis), anti-p53 (BD Transduction Laboratories), anti-acetylated histone H4 (Upstate Biotechnology), anti-FLAG (Sigma-Aldrich), and anti-phosphorylated p53 (Ser-15) antibody (Cell Signaling Technology).

We used the wild-type *RAD9 *plasmid and the phosphorylation-defective *RAD9 *mutants, kindly given from Dr. L. M. Karnitz [20]. To construct FLAG-tagged *RAD9 *expression plasmids, the DNAs were amplified by the PCR method with the advantage Clontaq system with high fidelity (Clontech) according to the manufacture's instruction, by using wild-type and *RAD9 *mutant vectors as templates and a set of primers (Table 1). The amplified products were separated by the electrophoresis, cut and purified with gel purification kit (Qiagen). After digestion with NotI and XbaI, the samples were ligated to the cloning site of pcDNA3.1-FLAG vector. WWP-Luc-*P21 *promoter vector, kindly given from Dr. B. Vogelstein [25], pcDNA-*TP53 *expression plasmid, kindly given from Dr. J. Yokota [41].

### Transfection

Plasmids were transfected with LipofectAMINE2000 according to manufacturer's instructions (Invitrogen). Double-stranded siRNAs for *hRAD9 *and *TP53 *(Santa Cruz Biotechnology) were transfected twice 24 h apart using TransIT-TKO transfection reagent (Mirus).

### Reverse transcription (RT)-PCR and real time PCR

RNA was extracted using ISOGEN protocol (Nippon Gene). First-strand cDNA was prepared from total RNA (5 μg) and oligo (dT) using the Superscript First-Strand Synthesis System (Invitrogen). The synthesized cDNA was amplified by PCR. The oligonucleotides used were shown in Table 1[42,43]. The PCR conditions were: for *P21*, an initial denaturation at 94°C for 1 min, followed by 37 cycles of 94°C for 8 sec, 53°C for 30 sec, and 72°C for 1 min; for *G3PDH*, the denaturation at 94°C for 1 min, followed by 28 cycles of 94°C for 10 sec, 60°C for 15 sec, and 72°C for 1 min. PCR products were separated by electrophoresis and visualized by ethidium bromide staining. The intensity of each band corresponding to PCR was quantified by densitometry analysis (Quantity One, BIO-RAD). The negative control without RTase showed no amplifications. Each experiment was repeated at least thrice. PCR-Southern blot analysis was performed as described [44], with minor modifications. Briefly, after four different cycles (24, 28, 32 and 36) of PCR, reactions (20 ul) in separate tubes were subjected to electrophoresis, transferred to nylon filter and were hybridized with [^32^P] dCTP-labeled *P21 *or *G3PDH *probe, which was amplified by RT-PCR. After washes, filter was exposed on x-ray film. For Real time RT-PCR assessment of *P21 *expression, total RNA was extracted and cDNA was synthesized. Primers and TaqMan probe were used for amplification and assessment according to the manufacture's instruction (Mm00432448_m1, Applied Biosystems).

### Protein study

For Western blotting, cells were extracted with lysis buffer [20 mM Tris-Hcl (PH 7.4), 1% Triton × 100, 10% glycerol, and 0.1 mM PMSF]. The cleared extracts were resolved by SDS-PAGE, and transferred to polyvinylidene difluoride membrane. Immunoblotting was performed by standards methods and signal was detected enhanced chemiluminescence system (ECL, Amarsham Biosciences). The intensity of each band was quantified by densitometry analysis (Quantity One, BIO-RAD). For immunoprecipitation (IP), cells were harvested with IP-lysis buffer [25 mM Tris-HCl (PH 7.5), 0.2% NP40, 250 mM NaCl, and 1 mM EDTA] and 500 μg of cell lysates, after being precleared with protein G-Sepharose beads, were incubated with 3 μg of specific antibody overnight. Antigen-antibody complex was immobilized on protein G-Sepharose beads, and washed five times in lysis buffer. Bound proteins were eluted by boiling and subjected to SDS-PAGE and immunoblotting.

### Promoter study

For luciferase reporter assay, transfected cells were cultured in a complete growth medium for 24 h and harvested, performed according to the manufacture's instruction (Promega). Each plasmids including empty expression vector were transfected with the same amounts (60 ng). Luciferase activity was measured on a Fluoroskan Ascent FL Luminometer (Thermo Labsystems Oy). As the internal control, each sample was co-transfected with pRL-TK, and the relative luciferase activity was figured out as the ratio of *Firefly *to *Renille *to adjust the tansfection rates. Each experiment was repeated at least thrice. Chromatin immunoprecipitation (ChIP) assay was performed using the Upstate Biotechnology kit. Briefly, ~1 × 10^6 ^cells were cross-linked with 1% formaldehyde, resuspended in 200 μL of SDS-lysis buffer, and sonicated to disrupt chromatin at an average length of 200 to 1,000 bp. After centrifugation, 20 μL of each supernatant were heated at 65°C for 4 h after the addition of 1 μL of 5 M NaCl, which was used as an input. The rest of the supernatant was added to 300 μL of ChIP dilution buffer containing 1 μg of either anti-Rad9 or anti-p53 antibody. After incubation at 4°C for 16 h, the immunoprecipitates were washed, eluted, heat treated, and digested with proteinase K. DNA was recovered by phenol/chloroform extraction and ethanol precipitation. We used one tenth (2 μL) of the final suspension for PCR using primers corresponding to different regions of the human *P21 *promoter. Primers used were shown in Table 1[33].

## Authors' contributions

Ka. I. performed experiments and participated in experimental design and writing of manuscript. H. I. conceived the study, reviewed and analyzed all data and drafted the manuscript. K. M. and M. M. performed quantitative RT-PCR. M. K. and K. Y. prepared the experimental materials and conceived the study. Y. M., H. N., Y. F. and Ke. I. participated in experimental design and writing of manuscript. All authors read and approved the final manuscript.

## Supplementary Material

Additional file 1 **UV-induced effect on the expression of *P21 *mRNA in MRC5 cell line**. MRC5 cells were exposed to UV at 20 J/m^2 ^and harvested 0, 1, and 3 hr after the treatment as indicated. Total RNA was extracted and RT-PCR was performed. The ratio of *P21 *mRNA to *G3PDH *is shown. Data represent the means ± SD of three independent experiments. Click here for file

Additional file 2 **Alteration of P21 mRNA expression in the knockdown experiment by *hRAD9 *or *TP53 *siRNA**. (A) Semi-quantitative RT-PCR after siRNA knockdown of hRad9 and p53. Cells were treated with *hRAD9 *(siRad9) or *TP53 *siRNA (siP53) and subjected to UV exposure. Cells were harvested and RNAs were extracted for RT-PCR with *P21 *primers. Samples were collected during each PCR cycle as indicated. The relative amount of *P21 *mRNA to *G3PDH *mRNA was measured with densitometry, and the data are shown. Data represent the means ± SD of three independent experiments. (B) Semi-quantitative determination of *P21 *mRNA using PCR-Southern blot analysis. (C) Real-time RT-PCR using *TP53*-deficient MEFs after siRNA knockdown of hRad9. Data represent the means ± SD of three independent experiments. Click here for file

Additional file 3 **UV-induced effect on the expression of *P21 *mRNA in TE-7 cell line**. (A) Western blots of lysates from TE-7 transfectants of wild-type *RAD9*, *RAD9*-9A mutant and *TP53 *plasmid, as indicated. Lysates were analyzed by Western blotting, probed with anti-p53, anti-phosphorylated p53 (Ser15), anti-p21 or anti-actin antibody. (B) Alteration of *P21 *mRNA expression in the transfection with wild-type *RAD9*, *RAD9*-9A mutant and *TP53 *plasmid. Cells were transfected with plasmids as indicated and subjected to UV exposure. The cells were harvested and RNAs were extracted for RT-PCR with *P21 *primers. The relative amount of *P21 *mRNA to *G3PDH *mRNA is shown. Data represent the means ± SD of three independent experiments. Click here for file

Additional file 4 **Interaction between hRad9 and p53 in MRC5**. Immunoprecipitation and a Western blot analysis were performed using cell lysate of MRC5. Anti-c-kit antibody was used as a negative control. Click here for file

## Figures and Tables

**Table 1:** Primers and Probes for Each Experiments

Names of Primers and Probes	Sequences
Target sequence of pBAsi-mU6 vector (TAKARA; Code: 3225)	5'-CCA CAC TCT TAG AGC AAG A-3'
PCR primers for wild-type and *RAD9 *mutants	5'-AAA AGC GGC CGC GCA TGA AGT GCC TGG TCA CGG G-3' and 5'-TTT TCT AGA TCA GCC TTC ACC CTC ACT GTC-3'
RT-PCR primers for *P21 *corresponding to an amplified product of 335 bp	5'-ACC CTC TCA TGC TCC AGG T-3' and 5'-CCT TGT TCC GCT GCT AAT CA-3'
RT-PCR primers for glyceraldehydes-3-phosphate dehydrogenase (*G3PDH*), to an amplified product of 451 bp product	5'-ACC ACA GTC CAT GCC ATC AC-3' and 5'-TCC ACC ACC CTG TTG CTG TA-3'
ChIP primers for human *P21 *downstream promoter	5'-GAG GTC AGC TGC GTT AGA GG-3' and 5'-TGC AGA GGA TGG ATT GTT CA-3'
ChIP primers for human *P21 *upstream promoter	5'-CCT ATG CTG CCT GCT TCC CAG GAA-3' and 5'-TAG CCA CCA GCC TCT TCT ATG CCA G-3'
