# Supplementary figures and images for: Rad9 modulates the P21WAF1 pathway by direct association with p53
Source: BMC Mol Biol. 2007 May 21;8:37. doi: 10.1186/1471-2199-8-37 (PMC1885445; doi:10.1186/1471-2199-8-37)

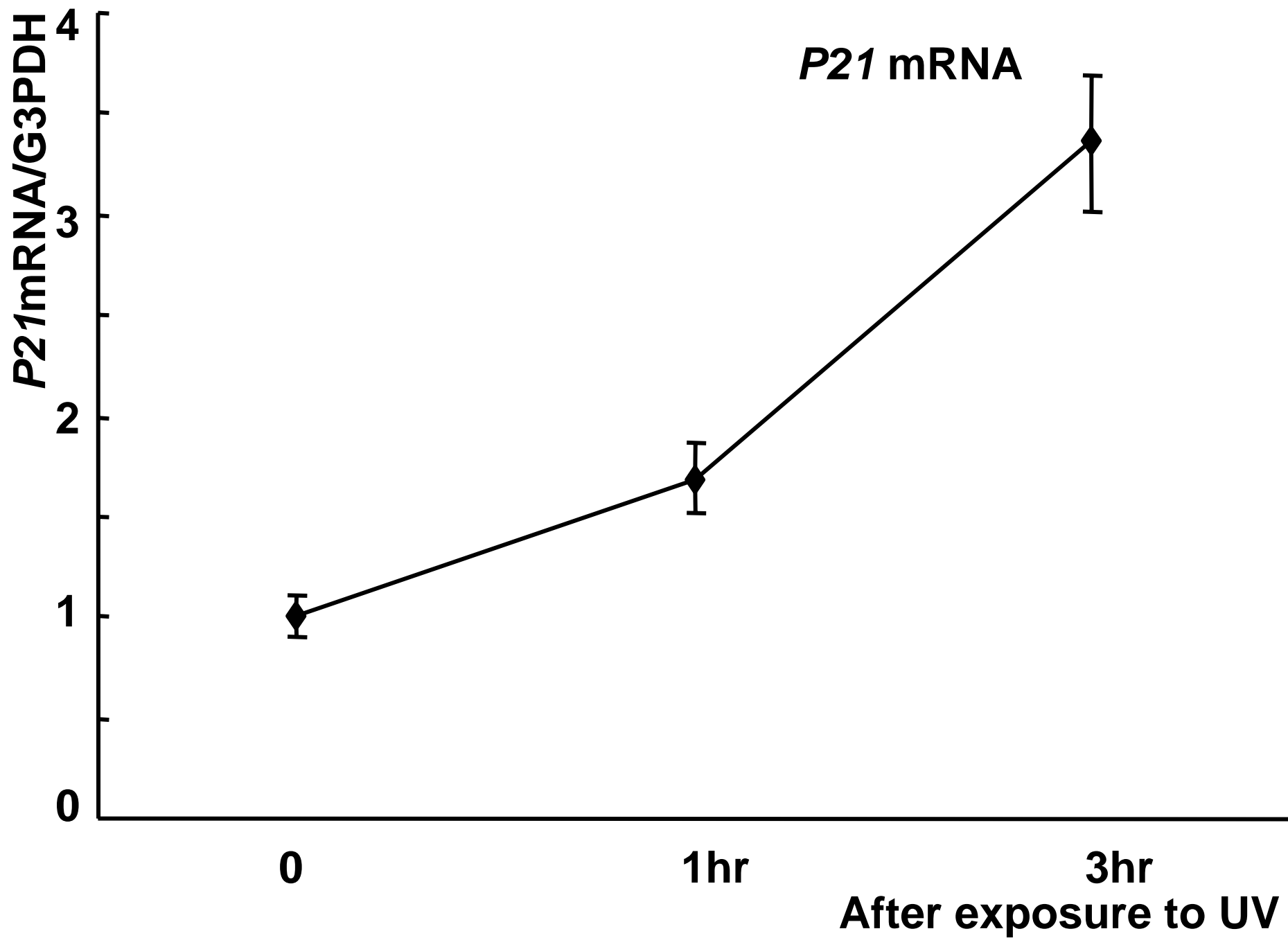

Supplement: Additional file 1 — UV-induced effect on the expression of P21 mRNA in MRC5 cell line. MRC5 cells were exposed to UV at 20 J/m2 and harvested 0, 1, and 3 hr after the treatment as indicated. Total RNA was extracted and RT-PCR was performed. The ratio of P21 mRNA to G3PDH is shown. Data represent the means ± SD of three independent experiments. [file 1471-2199-8-37-S1.pdf]

**A**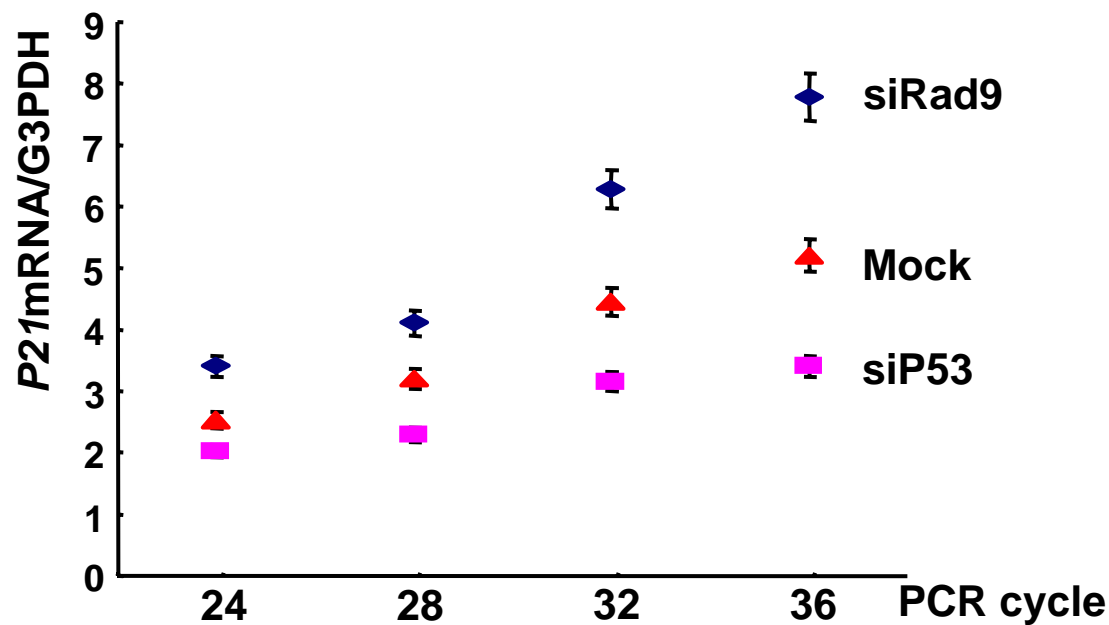**B**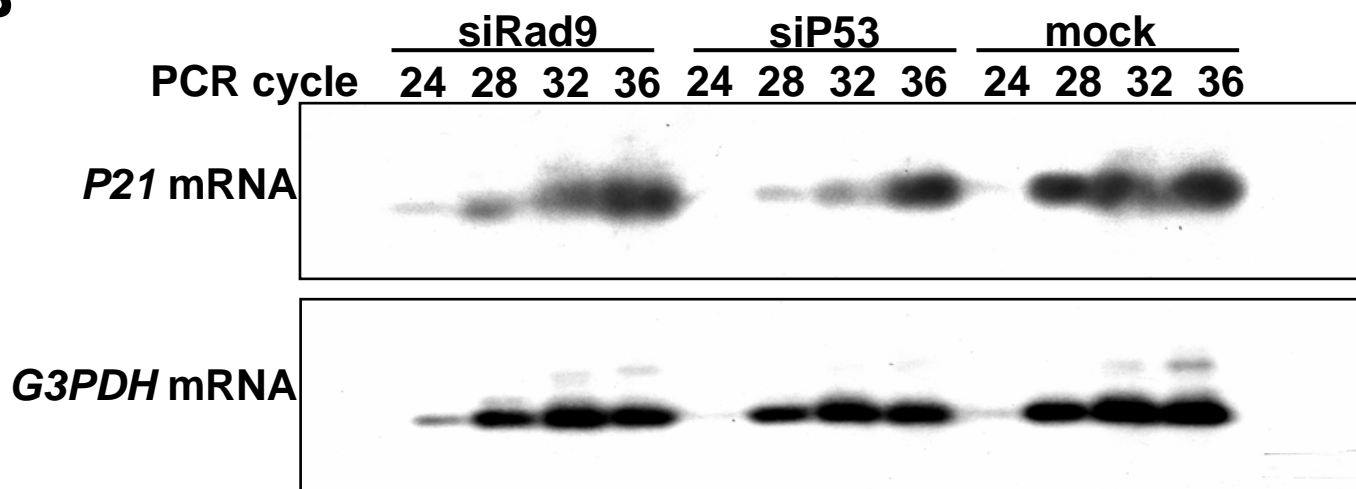**C**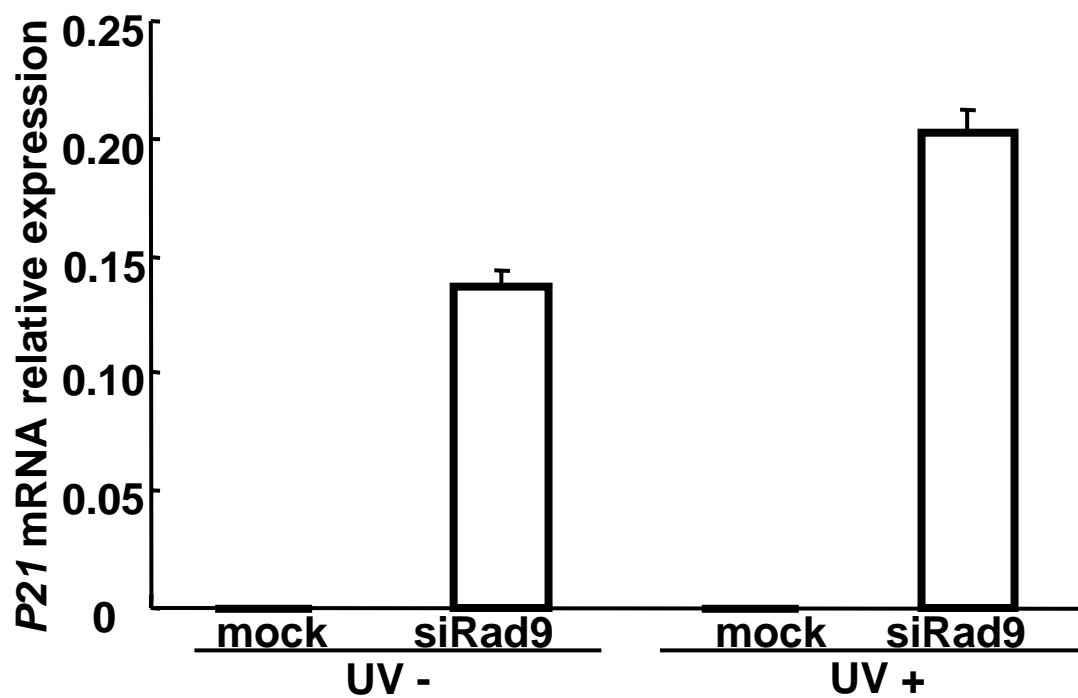

Supplement: Additional file 2 — Alteration of P21 mRNA expression in the knockdown experiment by hRAD9 or TP53 siRNA. (A) Semi-quantitative RT-PCR after siRNA knockdown of hRad9 and p53. Cells were treated with hRAD9 (siRad9) or TP53 siRNA (siP53) and subjected to UV exposure. Cells were harvested and RNAs were extracted for RT-PCR with P21 primers. Samples were collected during each PCR cycle as indicated. The relative amount of P21 mRNA to G3PDH mRNA was measured with densitometry, and the data are shown. Data represent the means ± SD of three independent experiments. (B) Semi-quantitative determination of P21 mRNA using PCR-Southern blot analysis. (C) Real-time RT-PCR using TP53-deficient MEFs after siRNA knockdown of hRad9. Data represent the means ± SD of three independent experiments. [file 1471-2199-8-37-S2.pdf]

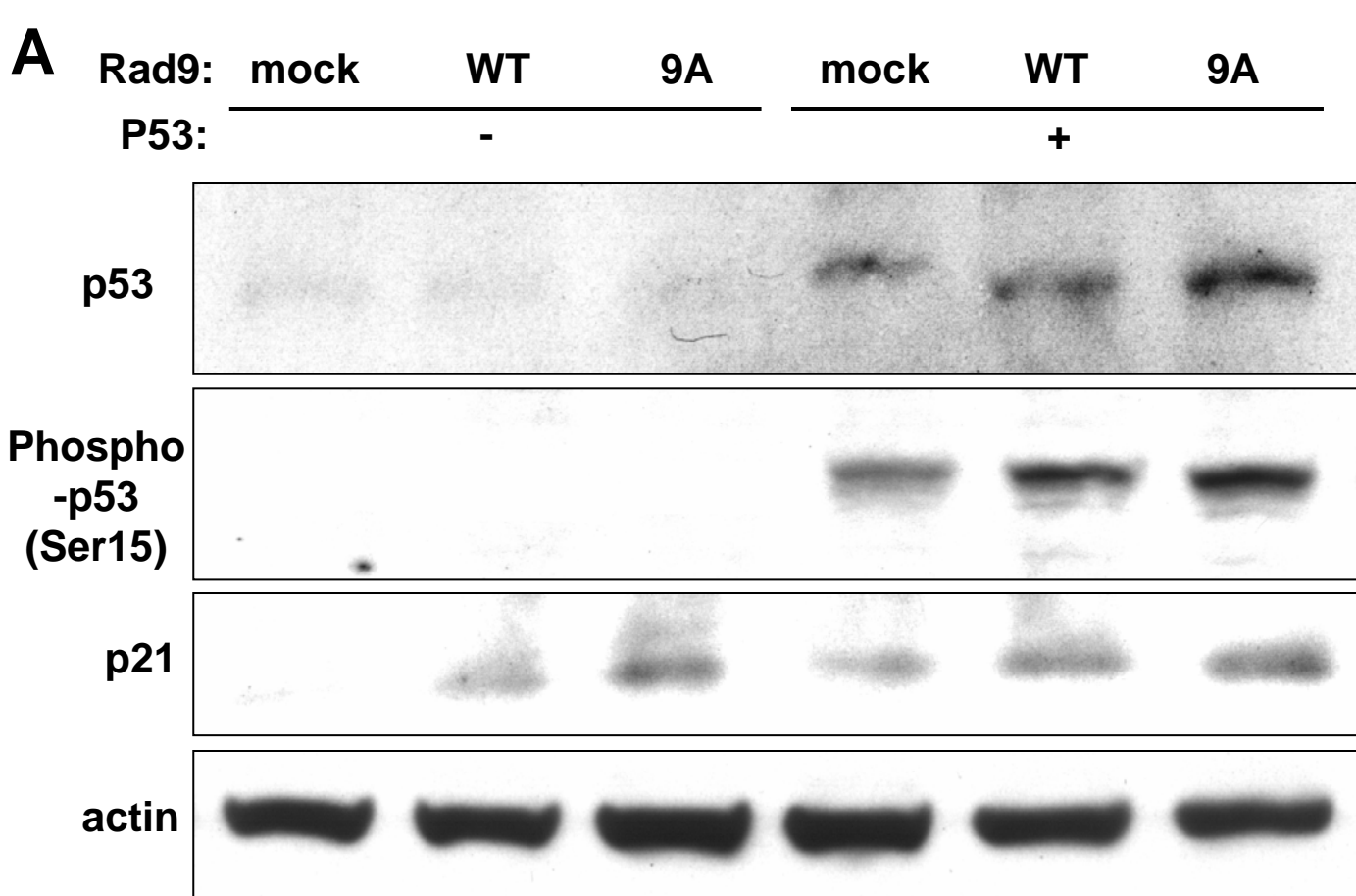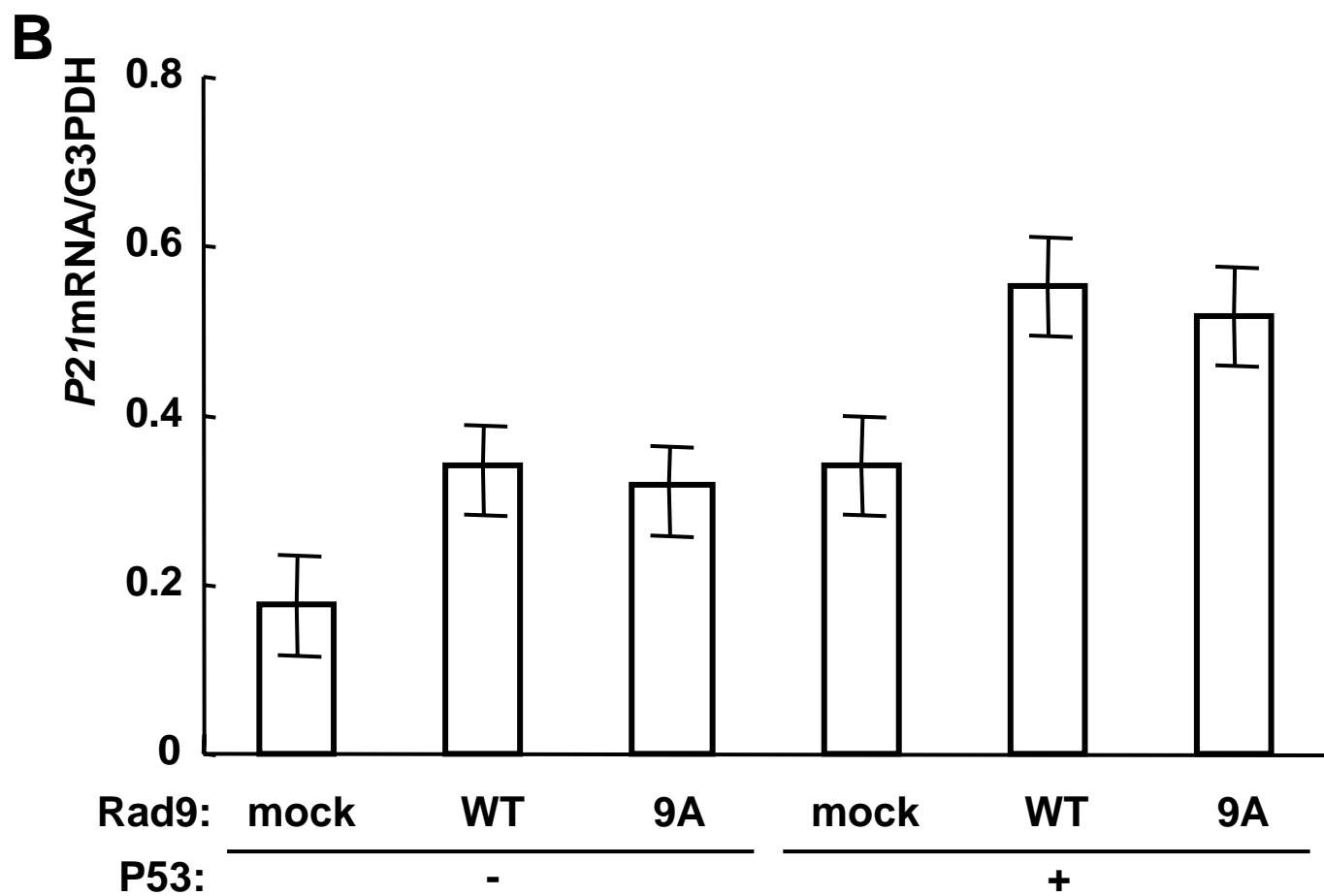

Supplement: Additional file 3 — UV-induced effect on the expression of P21 mRNA in TE-7 cell line. (A) Western blots of lysates from TE-7 transfectants of wild-type RAD9, RAD9-9A mutant and TP53 plasmid, as indicated. Lysates were analyzed by Western blotting, probed with anti-p53, anti-phosphorylated p53 (Ser15), anti-p21 or anti-actin antibody. (B) Alteration of P21 mRNA expression in the transfection with wild-type RAD9, RAD9-9A mutant and TP53 plasmid. Cells were transfected with plasmids as indicated and subjected to UV exposure. The cells were harvested and RNAs were extracted for RT-PCR with P21 primers. The relative amount of P21 mRNA to G3PDH mRNA is shown. Data represent the means ± SD of three independent experiments. [file 1471-2199-8-37-S3.pdf]

**IP**

**Input**

**p53**

**Rad9**

**control**

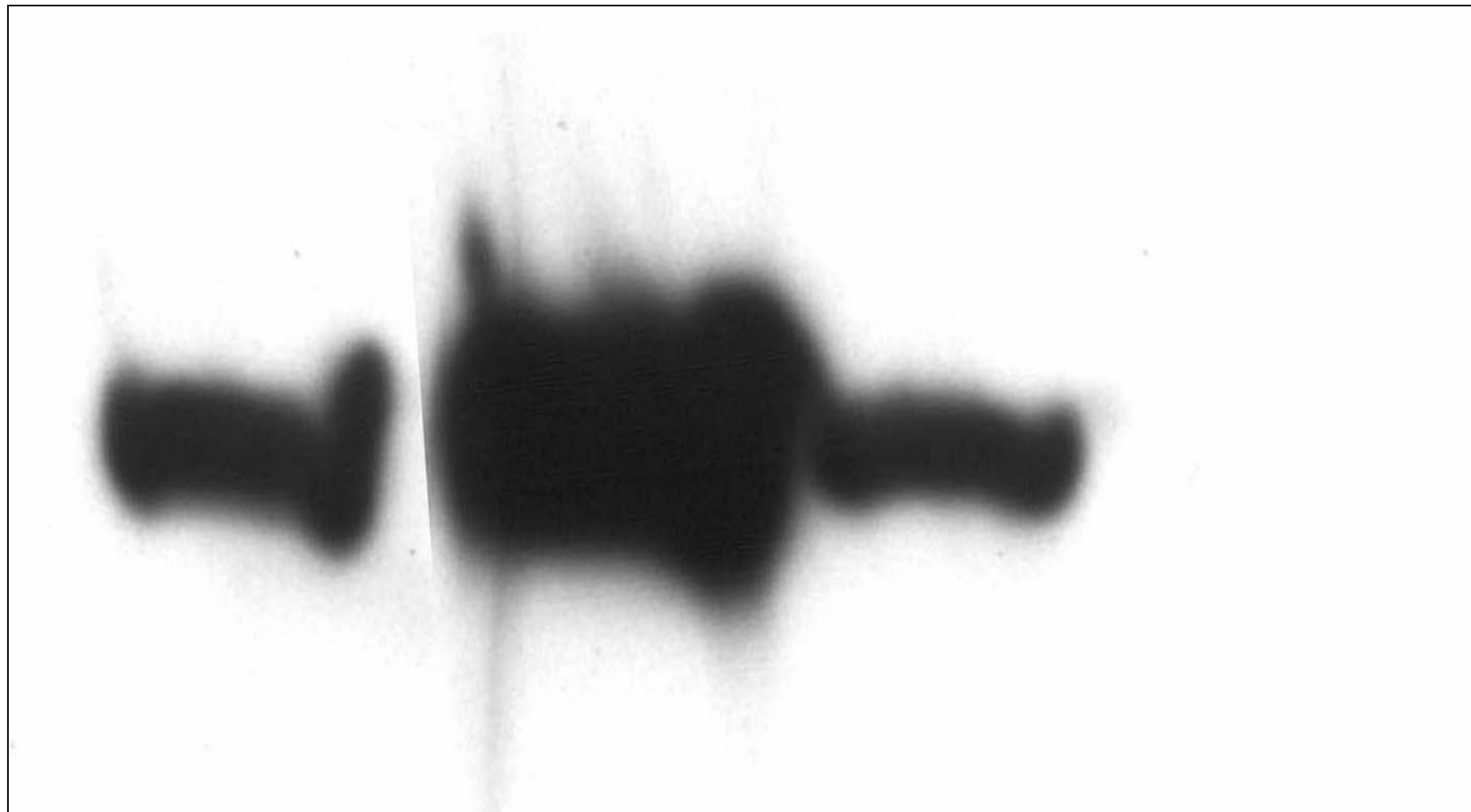

**WB: p53**

Supplement: Additional file 4 — Interaction between hRad9 and p53 in MRC5. Immunoprecipitation and a Western blot analysis were performed using cell lysate of MRC5. Anti-c-kit antibody was used as a negative control. [file 1471-2199-8-37-S4.pdf]
